# Spin Readout Techniques of the Nitrogen-Vacancy Center in Diamond

**DOI:** 10.3390/mi9090437

**Published:** 2018-08-30

**Authors:** David A. Hopper, Henry J. Shulevitz, Lee C. Bassett

**Affiliations:** 1 Quantum Engineering Laboratory, Department of Electrical and Systems Engineering, University of Pennsylvania, Philadelphia, PA 19104, USA; dhop@sas.upenn.edu (D.A.H.); shuhenry@seas.upenn.edu (H.J.S.); 2 Department of Physics and Astronomy, University of Pennsylvania, Philadelphia, PA 19104, USA

**Keywords:** nitrogen-vacancy center, spin readout, quantum information, sensing

## Abstract

The diamond nitrogen-vacancy (NV) center is a leading platform for quantum information science due to its optical addressability and room-temperature spin coherence. However, measurements of the NV center’s spin state typically require averaging over many cycles to overcome noise. Here, we review several approaches to improve the readout performance and highlight future avenues of research that could enable single-shot electron-spin readout at room temperature.

“The only thing you can do easily is be wrong, and that’s hardly worth the effort.”

—Norton Juster, *The Phantom Tollbooth*

## 1. Introduction

Recent, rapid advances in creating, detecting, and controlling quantum-mechanical states in engineered systems heralds the beginning of the quantum-information era. A diverse set of physical platforms, including superconducting circuits [1], cold ions [2], integrated photonics [3], and spins in semiconductors [4], have enabled progress toward fault-tolerant quantum computation, quantum-secure communication systems, and unparalleled sensing technologies. Nevertheless, most platforms remain in the early engineering stages and face substantial technical challenges. A common challenge, and critical criterion for scalable quantum information processing [5], is reliably measuring the quantum state. The issue of precision measurement is one of the oldest and most subtle aspects of quantum theory, and it is arguably the most essential for many practical applications. Several authors have reviewed general considerations for quantum measurements [6,7]. Here, we focus on the problem as applied to the nitrogen-vacancy (NV) center in diamond, which has emerged as a compelling solid-state qubit for a wide range of quantum technologies.

Point defects in wide-bandgap semiconductors are analogous to molecules trapped within a crystalline host. A small subset of these point defects functions as qubits with optical addressability, exceptional spin coherence properties, and room-temperature operation [8]. The diamond nitrogen-vacancy (NV) center is the prototypical defect spin qubit, and the most intensely studied [9]. A truly versatile platform, the NV center has been utilized for designing quantum memories [10,11,12]; addressing individual nuclear spins [13,14,15]; engineering nanoscale sensors of magnetism [16], proteins [17], and chemicals [18]; exploring hybrid quantum mechanical systems [19]; and testing the fundamental principles of quantum mechanics through loophole free violations of Bell’s inequality [20]. In the course of these investigations, several techniques have been developed to measure the NV center’s spin state that offer certain advantages for specific circumstances. Here, we review the leading techniques for NV spin readout by presenting the physical mechanisms, discussing the state-of-the-art and considering the potential for further improvement. Due to the breadth of NV research, we direct readers to detailed reviews on quantum sensing [21], NV magnetometry [22], nanodiamond sensing [23], and nanophotonics in diamond [24] for an overview of these application areas.

The review is organized as follows: Section 2 overviews several spin-readout performance metrics commonly used in the community; Section 3 introduces the traditional approach to spin readout using photoluminescence (PL); Section 4 discusses recent efforts to improve photon collection efficiency; Section 5 considers how altering the excited state lifetime affects spin readout; Section 6 introduces the resonance-fluorescence technique for single-shot spin readout at low temperature; Section 7 describes how coupled nuclear spins can improve the electron-spin readout; Section 8 overviews protocols for spin-to-charge conversion; Section 9 discusses recent advances in measuring the spin state through photocurrent; Section 10 explains how accounting for measurement overhead can improve the time-averaged signal-to-noise ratio and sensitivity; Section 11 discusses the use of real-time signal processing; Section 12 considers the potential for combining different techniques; Section 13 summarizes the review and provides an outlook on the future of NV applications enabled by maturing readout techniques.

## 2. Quantifying Readout Performance

Various metrics are used by the NV community to quantify readout performance, each with intuitive advantages for specific applications. As we show in this section, the common metrics all relate to the signal-to-noise ratio (SNR) of the measurement, which provides a useful basis to compare different readout techniques. We consider projective measurements where the goal is to distinguish between two quantum states, |0〉 and |1〉. Therefore, we define the SNR for a differential measurement,

(1) $$\mathrm{SNR}=\frac{\langle S_{0}\rangle -\langle S_{1}\rangle }{\sqrt{\sigma_{0}^{2}+\sigma_{1}^{2}}},$$

where $\langle S_{i}\rangle$ is the mean signal for a single measurement of spin state |i〉, and $\sigma_{i}$ is the associated noise. Classical signal processing [25] and superconducting qubits [26] both employ an analogous definition of differential SNR. In the following subsections, we discuss common optical-detection signals and their associated SNR, relate the SNR to other spin-readout metrics, and discuss how to include averaging over multiple experimental cycles.

### 2.1. Photon Summation

In many situations, the signal is simply the number of photons detected in a fixed readout cycle. In this case, Equation (1) takes the form:

(2) $$\mathrm{SNR}=\frac{\alpha_{0}-\alpha_{1}}{\sqrt{\alpha_{0}+\alpha_{1}}},$$

where $\alpha_{i}$ is the mean number of detected photons for a single measurement of spin state |i〉. Here, we assume $\alpha_{0}>\alpha_{1}$ and that the noise in each signal is dominated by photon shot noise, with variance $\sigma_{i}^{2}=\alpha_{i}$. The SNR is related to the dimensionless contrast between the two signals,

(3) $$C=\left(1-\frac{\alpha_{1}}{\alpha_{0}}\right),$$

such that the photon-summation SNR can be recast as:

(4) $$\mathrm{SNR}=\sqrt{\alpha_{0}}\times \frac{C}{\sqrt{2-C}}.$$

This formulation clearly separates the SNR’s dependence on photon collection efficiency and spin contrast. Note that our definition of *C* differs from the related metric used by some authors, which we term the visibility, $V\;=\;\left(\alpha_{0}-\alpha_{1}\right)/\left(\alpha_{1}+\alpha_{0}\right)$. Adding to potential confusion, the dimensionless parameter *C* defined in the seminal work by Taylor et al. [27] is neither the contrast, nor the visibility, but is rather the inverse of the spin-readout noise, discussed in Section 2.3. For the case of NV centers, it is natural to define the contrast as in Equation (3) since $\alpha_{0}$ is related to the optically pumped initial spin state and often appears in defining the normalized PL, $S/\alpha_{0}$. For an NV center in bulk diamond, typically C≈0.3 using the traditional PL-based readout approach. In the limit of perfect contrast (C=1), the photon-summation SNR is limited by shot noise alone.

### 2.2. Thresholding

If many photons are detected during a single measurement cycle, the photon summation technique becomes less efficient than assigning a discrete outcome based on a threshold condition [28]. In this scenario, the signal is modeled by the sum of two photon probability distributions (typically Poissonian or Gaussian) with different means. A threshold value is selected to distinguish between the two distributions, resulting in a binomial random variable specifying the outcome zero or one. For example, suppose the |0〉 state generates a detected number of photons that exceeds the threshold (yielding binary S=1) with probability $p_{0|0}$, whereas |1〉 generates a detection event that exceeds the threshold with probability $p_{0|1}$. Here, $p_{0|0}$ is the true positive rate, implying a false negative rate $\epsilon_{0}=1-p_{0|0}$, whereas $\epsilon_{1}=p_{0|1}$ is the false positive rate. The readout fidelity, a measure of the confidence in a given measurement outcome, is defined in terms of these two error rates as [28,29]:

(5) $$F=1-\frac{1}{2}\left(\epsilon_{0}+\epsilon_{1}\right).$$

The fidelity takes values between 50% and 100%, assuming an optimal threshold condition has been selected.

The binomial nature of thresholded readout facilitates the direct evaluation of the signal mean and variance for an initial spin state |i〉,

(6) $$\langle S_{i}\rangle =p_{0|i}$$

(7) $$\sigma_{i}^{2}=p_{0|i}\left(1-p_{0|i}\right),$$

from which we can calculate the corresponding differential SNR directly from Equation (1):

(8) $$\mathrm{SNR}=\frac{p_{0|0}-p_{0|1}}{\sqrt{p_{0|0}\left(1-p_{0|0}\right)+p_{0|1}\left(1-p_{0|1}\right)}}.$$

Assuming symmetric error probabilities, $\epsilon_{0}=\epsilon_{1}$, Equation (8) takes the simplified form:

(9) $$\mathrm{SNR}=\frac{2F-1}{\sqrt{2F(1-F)}}.$$

This formulation provides a standard criterion, sometimes quoted in the literature, for determining whether a quantum state readout is single-shot; a readout fidelity F>79% corresponds to an SNR > 1.

Oftentimes, the measured value of F is less than would be predicted from the ideal SNR [30,31,32]. This discrepancy stems from backaction (unwanted state changes during the measurement) and also potentially from improper state initialization. We will discuss these issues below in the context of different readout techniques.

### 2.3. Spin-Readout Noise

In a quantum sensor, the environmental state is mapped onto the qubit state such that the information is contained in a population difference, resulting in a stochastic signal whose mean is given by:

(10) $$\langle S\rangle =\cos^{2}\left(\frac{\theta }{2}\right)\langle S_{0}\rangle +\sin^{2}\left(\frac{\theta }{2}\right)\langle S_{1}\rangle .$$

Here, the angle θ depends on some external field (resulting, for example, from free evolution under an external magnetic field, *B*, such that θ∝B). The minimum resolvable angular shift, δθ, corresponds to the situation when the change in signal exceeds the noise, $\sigma_{S}$. Mathematically,

(11) $$\delta \theta =\frac{\sigma_{S}}{\left|\frac{\partial \langle S\rangle }{\partial \theta }\right|}.$$

For an ideal measurement, $\langle S_{0}\rangle =0$, $\langle S_{1}\rangle =1$, and $\sigma_{0}=\sigma_{1}=0$. However, the ideal measurement still exhibits noise due to the stochastic projection of qubit states. This projection noise is the basis for the standard quantum limit (SQL) for detecting angular shifts in a single measurement. Since projection is a binomial process, the variance of the signal depends on θ, similarly to the case of Equation (7) for thresholded measurements:

(12) $$\sigma_{\mathrm{SQL}}=\sqrt{p_{0}\left(\theta \right)\left[1-p_{0}\left(\theta \right)\right]}=\frac{1}{2}\sin\left(\theta \right).$$

Since the change in signal varies identically,

(13) $$\frac{\partial \langle S_{\mathrm{SQL}}\rangle }{\partial \theta }=\frac{1}{2}\sin\left(\theta \right),$$

the SQL for a single-shot measurement is a constant angle given by $\delta \theta_{\mathrm{SQL}}\equiv \theta_{0}=1$ radian.

Given this fundamental limit, it is illustrative to define a parameter that quantifies the effect of realistic, imperfect measurements. The spin-readout noise,

(14) $$\sigma_{R}\equiv \frac{\sigma_{S}}{\left|\frac{\partial \langle S\rangle }{\partial \theta }\right|\theta_{0}},$$

is a dimensionless quantity ≥1, where a value $\sigma_{R}=1$ signifies a measurement at the SQL [27,33]. The minimum experimentally-resolvable angular shift is then given by:

(15) $$\delta \theta =\theta_{0}\sigma_{R}.$$

This formulation explicitly separates the resolution limit into two categories: the quantum mechanical noise ($\theta_{0}$) and experimental noise ($\sigma_{R}$). A related metric, also called the readout fidelity by some authors [17,27], is simply the inverse, $\sigma_{R}^{-1}$. This definition of readout fidelity spans the range (0,1], where unity indicates an ideal measurement, and it differs fundamentally from the traditional definition of quantum readout fidelity (Equation (5)). We use the traditional definition for F in the remainder of this work.

### 2.4. Averaging

The preceding discussion concerns single-shot readout of individual qubits. In many cases, it is advantageous to repeat the measurement (including, usually, a full experimental cycle of initialization and coherent evolution) many times in order to identify small signals. This is especially true when the single-shot SNR is well-below unity. Assuming independent trials, the SNR formulation provides a simple means for calculating the time-averaged SNR, namely,

(16) $$\langle \mathrm{SNR}\rangle =\sqrt{N}\times \mathrm{SNR},$$

where 〈〉 signifies the time-average and *N* is the number of measurements. The parameter *N* can account for measurements averaged in space (for ensembles of identical qubits) or time (for repeated measurements). In the remainder of this review, we consider especially the case of time-averaging, where *N* is related to the total integration time, and Equation (16) allows for the direct comparison of different measurement techniques while accounting for the overhead from varying measurement durations. Especially for sensing applications, it bears remembering that qubit ensembles offer an additional improvement that scales with the square root of the ensemble size.

### 2.5. Sensitivity

Sensors generally aim to acquire as much information as possible about an environmental state before it changes. Accordingly, we must quantify the tradeoff between signal amplitude and measurement bandwidth. Usually, signals are averaged over many experimental cycles, and it is useful to define the field sensitivity,

(17) $$\eta =f\left(\theta_{0}\right)\sigma_{R}\sqrt{\tau },$$

where the function $f(\theta_{0})$ relates the SQL to a particular field amplitude, and τ is the time it takes to perform a single measurement cycle, including initialization, operation, and readout. The sensitivity has dimensions of $\left[\mathrm{field}\;\mathrm{amplitude}\right]\;\cdot \;{\mathrm{Hz}}^{-1/2}$, and the minimum resolvable field can be estimated by dividing η by the square root of total integration time. Barring additional noise sources or instability in the field to be measured, arbitrarily low fields can be resolved by integrating for longer times.

Two common sensing applications are the detection of dc and ac magnetic fields [21,27]. For the case of dc magnetic fields, the field amplitude is mapped onto a quantum phase difference using a Ramsey sequence, with a corresponding SQL given by:

(18) $$f_{B_{\mathrm{dc}}}\left(\theta_{0}\right)=\frac{\hbar }{g\mu_{B}T_{2}^{*}}\theta_{0},$$

where *g* is the gyromagnetic ratio, $\mu_{B}$ is the Bohr magneton, and $T_{2}^{*}$ is the inhomogeneous spin dephasing time. Dropping the factor $\theta_{0}=1$, the corresponding sensitivity is:

(19) $$\eta_{B_{\mathrm{dc}}}=\frac{\hbar }{g\mu_{B}}\sqrt{\frac{T_{2}^{*}+t_{I}+t_{R}}{{\left(T_{2}^{*}\right)}^{2}}}\sigma_{R},$$

where $t_{I}+t_{R}$ is the time required to initialize and read out the spin state, which will be referred to as measurement overhead in this review. Similarly, oscillating magnetic fields are detected by implementing a Hahn echo or dynamical decoupling sequence to accumulate phase. In this case, the ac field resolution is:

(20) $$f_{B_{\mathrm{ac}}}\left(\theta_{0}\right)=\frac{\pi \hbar }{2g\mu_{B}T_{2}}\theta_{0},$$

where $T_{2}$ is the homogeneous spin dephasing time, and the corresponding sensitivity is:

(21) $$\eta_{B_{\mathrm{ac}}}=\frac{\pi \hbar }{2g\mu_{B}}\sqrt{\frac{T_{2}+t_{I}+t_{R}}{{\left(T_{2}\right)}^{2}}}\sigma_{R}.$$

In general, both $\sigma_{R}$ and η depend on the average value of θ at which the measurement is performed. In most cases, however, the optimum conditions for sensing are very close to θ=π/2. Making this assumption, we derive the following analytic expressions for the spin-readout noise for the cases of photon summation,

(22) $$\sigma_{R}^{\mathrm{Photon}}=\sqrt{1+2\frac{\alpha_{0}+\alpha_{1}}{{\left(\alpha_{0}-\alpha_{1}\right)}^{2}}},$$

and for thresholding,

(23) $$\sigma_{R}^{\mathrm{Threshold}}=\sqrt{1+2\frac{p_{0|0}\left(1-p_{0|0}\right)+p_{0|1}\left(1-p_{0|1}\right)}{{\left(p_{0|0}-p_{0|1}\right)}^{2}}}.$$

Derivations are included in Appendix A. In both cases, the spin-readout noise is directly related to the differential SNR, following the general expression:

(24) $$\sigma_{R}=\sqrt{1+\frac{2}{{\mathrm{SNR}}^{2}}}.$$

The combination of Equation (24) with Equation (17) provides a general approach to calculate the sensitivity for all spin-readout techniques covered in this review, while also accounting for variable readout durations where the SNR further becomes a function of τ (discussed in Section 8).

### 2.6. Summary

Particular applications benefit from different aspects of the spin-readout metrics described in the previous subsections. For example, quantum algorithms generally demand single-shot readout with small error probabilities. Therefore, readout fidelity is the most informative choice. Magnetometry and sensing applications, on the other hand, usually rely on time-averaging and are inherently subject to the standard quantum limit; in this case, spin-readout noise is the most illuminating metric. Each of these metrics can be related to the SNR, which serves as a useful basis of comparison across multiple techniques. Table 1 summarizes the three metrics discussed in this section and their relation to SNR.

In some situations, a critical experimental design consideration is whether to use photon summation or thresholding. To decide, we can compare the thresholding SNR (Equation (8)) to the photon summation SNR (Equation (2)) and choose the higher value. Typically, thresholding becomes more efficient when one of the spin states produces >1 photon in a measurement and the contrast exceeds 50%. We hope that the connections between these metrics and various measurement techniques described in the following sections will aid in selecting the optimal approach for future applications.

## 3. Traditional Spin Readout

The NV center’s intrinsic, spin-dependent PL facilitated the first room-temperature quantum control experiments with single spins [34,35]. Simply by counting the PL photons emitted in the first ∼ 300 ns of optical illumination and averaging over many cycles, the NV center’s ground-state spin projection can be inferred. This technique, here called traditional PL readout, is still widely used in research and applications due to its simple experimental implementation. This section outlines the physical mechanisms that underlie traditional PL readout, as well as some of the technique’s limitations.

The negatively-charged NV center is a point defect with $C_{3v}$ symmetry that exhibits isolated electronic states deep within the diamond’s band gap including a paramagnetic triplet ground state [9]. The $C_{3v}$ symmetry axis points along any of the 〈111〉 crystallographic axes, connecting the substitutional nitrogen and adjacent vacancy. The broken inversion symmetry leads to a zero-field energy splitting between the ground state’s $m_{s}=0$ and $m_{s}=\pm 1$ spin sub-levels ( 2.87 GHz at room temperature, with energies here and throughout given in frequency units), and a dc magnetic field applied along the defect’s symmetry axis further splits the $m_{s}=\pm 1$ levels such that individual transitions can be addressed using spin resonance techniques. This yields the commonly-used qubit manifolds, encompassing the $m_{s}=0$ state and one of the $m_{s}=\pm 1$ projections. Diamond’s low nuclear-spin density and weak spin-phonon coupling allow for the NV center’s spin coherence to reach milliseconds at room temperature [36]. The long spin coherence times allow for the detection of weak magnetic fields [27], including those associated with proximal nuclear [13] and electron [37] spins, enabling the realization of multi-qubit quantum registers [38,39].

**Figure 1:**
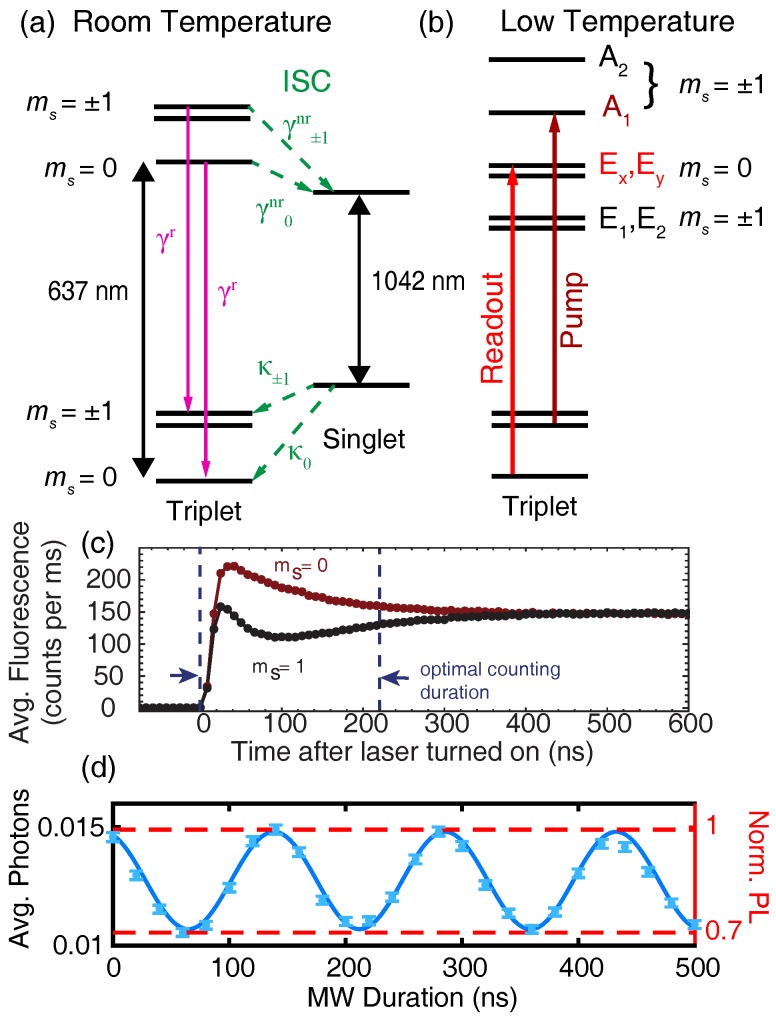
The diamond NV center. (**a**) Room temperature electronic structure. Solid lines indicate radiative transitions (with corresponding rate $\gamma^{r}$), and dashed lines represent nonradiative intersystem crossing (ISC) transitions (with rates $\gamma_{i}^{r}$ and $\kappa_{i}$ for the excited and ground-state spin projection *i*, respectively). Solid black arrows represent the zero phonon lines of the triplet and singlet manifolds. (**b**) Low temperature electronic structure of the nitrogen-vacancy (NV) center triplet manifold. Individual transitions used for spin pumping and resonant readout are indicated. (**c**) Room temperature transient fluorescence response for the spin states $m_{s}=0,1$ produced by 532 nm illumination. The optimal counting duration is indicated by the dashed vertical lines. Reprinted with permission from [42], Optical Society of America. (**d**) Rabi nutations of the ground-state spin at room temperature, measured using traditional PL readout for an NV center beneath a planar diamond surface, with an NA = 0.9 objective. The left and right axes plot the average detected photons per measurement and normalized PL, respectively. The solid curve is a fit to the data.

Under visible illumination (typically 532 nm), the NV center emits PL in its ≈650– 750 nm phonon sideband (PSB) whose intensity depends on the ground-state spin projection. Physically, spin-dependent PL arises from spin-orbit interactions within the intersystem crossing (ISC) that couples the triplet and singlet manifolds [40,41]. As shown in Figure 1a, the excited-state triplet levels can undergo radiative transitions back to the ground state or nonradiatively decay into the meta-stable singlet manifold. The total decay rate of the excited state spin projection |i〉 is given by the sum of these two rates, namely:

(25) $$\gamma_{i}=\gamma^{r}+\gamma_{i}^{\mathrm{nr}}.$$

The radiative rate, $\gamma^{r}$, is essentially spin independent, whereas the nonradiative rates, $\gamma_{i}^{\mathrm{nr}}$, depend strongly on the spin projection due to the spin-dependent ISC. Recent studies concluded that $\gamma_{\pm 1}^{\mathrm{nr}}\approx 10\gamma_{0}^{\mathrm{nr}}$ [41,42,43]. This difference produces a transient response to illumination that is drastically different depending on the initial projection of the ground-state spin.

Assuming the NV center is illuminated with an optical excitation rate similar to $\gamma^{r}$ (i.e., close to optical saturation, which is generally ideal for traditional PL readout), a spin population initially in $m_{s}=\pm 1$ is shelved into the singlet manifold within only a few optical cycles of the triplet states, whereas a population in $m_{s}=0$ continues to cycle and produce PL. This spin-dependent PL contrast is the essence of traditional readout. The contrast is short-lived, however; it vanishes after about 300 ns as the singlet population decays back to the triplet ground-state [44], and the system reaches a steady state (Figure 1c). Taking into account the spin selectivity of both the triplet-to-singlet and singlet-to-triplet ISC (the latter is less spin selective than the former), the resulting ground-state spin population after the illumination is switched off is ≈80% polarized into the $m_{s}=0$ sub-level [9,45,46]. This optically pumped pseudo-pure state generally serves as the initialized |0〉 state for subsequent quantum experiments, while one of the $m_{s}=\pm 1$ state serves as the |1〉 state.

Figure 1d shows a typical example of room-temperature Rabi nutations for a single NV center in bulk diamond, with the data plotted in terms of both the average number of photons detected per shot and the corresponding normalized PL. The spin contrast is *C* = 30%, and the confocal setup collects $\alpha_{0}$ = 0.015 photons on average from the |0〉 spin state, using an NA = 0.9 air objective to image an NV center ≈ 4 μm beneath a planar diamond surface with a saturated count rate of 50 kCts/s under continuous-wave 532 nm illumination. Using Equation (2), the corresponding single-shot SNR is 0.03, and Equation (16) implies that more than $10^{5}$ repeats are required to achieve 〈SNR〉=10. Each point in Figure 1d consists of $4\times 10^{5}$ repeats. In many applications, such averaging places severe limitations on performance and efficiency. In the remaining sections, we compare several alternative readout techniques to this standard, accounting for experimental variations in collection efficiency where possible.

## 4. Maximizing Photon Collection Efficiency

The NV’s optical addressability in a solid-state host material provides both technological opportunities and formidable engineering challenges. Due to the high refractive index of diamond (n≈2.4), total internal reflection at diamond-air interfaces severely limits the collection efficiency; even assuming an air objective with NA = 0.95, a maximum fraction of only 4% of emitted photons can be extracted through a planar (100)-oriented surface [47]. Since the spin-readout noise is dominated by the Poisson statistics associated with counting photons, collection efficiency improvements that increase $\alpha_{0}$ boost the single-shot SNR according to $\sqrt{\alpha_{0}}$ (Equation (4)) and reduce the averaging requirements according to $N\propto 1/\alpha_{0}$. This section considers strategies for improving the collection efficiency of NV centers within bulk diamond.

### 4.1. Crystal Alignment

The NV center’s optical dipoles are oriented perpendicularly to the symmetry axis connecting the nitrogen atom to the vacancy. Since the symmetry axis points along a crystalline 〈111〉 direction, aligning the optical axis perpendicularly to the corresponding {111} face maximizes optical absorption and emission. However, the (100) orientation of most commercially available synthetic diamonds misaligns the NV’s symmetry axis by 55 ${}^{\circ }$ from the optical axis. Using a 100x, NA = 0.9 air objective, Jamali et al. [48] showed that proper alignment of the dipole and optical axes results in a 65% increase in collected photons, corresponding to an SNR increase of ≈1.3. Although the production of (111)-faced diamonds is traditionally a laborious and expensive process, recent developments of laser-nucleated-cleaving techniques [49] provide an attractive alternative. In this technique, a series of laser pulses is used to nucleate and propagate cleaves along desired (111) planes within a standard (100)-faced diamond, resulting in large, flat, (111)-faced plates even without any polishing. Ideally-oriented NVs can then be produced using standard electron-irradiation or nitrogen implantation techniques, followed by annealing. Furthermore, recent studies have shown that growth of diamond along 〈111〉 directions can yield deterministically-oriented NVs with the optimum alignment perpendicular to the (111) surface [50,51,52,53,54].

### 4.2. Photonic Structures

**Figure 2:**
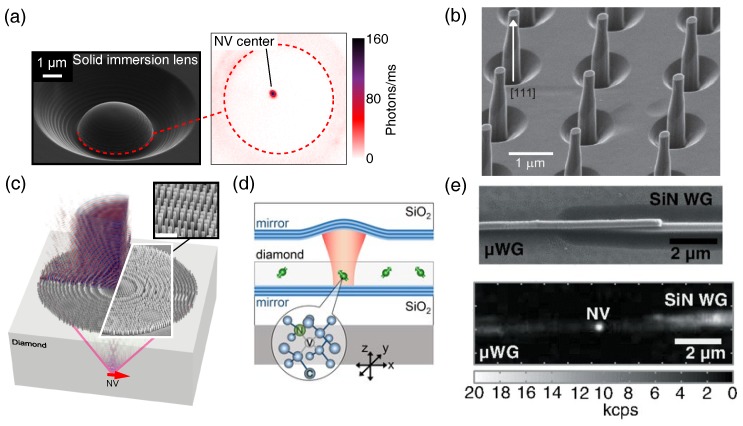
Photonic devices for improving collection efficiency (**a**) Scanning electron micrograph (SEM) of a solid immersion lens fabricated around an NV center. Inset: confocal PL image. (**b**) Diamond nanopillar array fabricated on a [111]-oriented diamond. Source: Neu et al. [58]. (**c**) Metalens fabricated above an NV center to act as an immersion objective. Inset: SEM of nanopillar metalens elements. Source: Grote et al. [57]. (**d**) Schematic of diamond membrane embedded in an open micro-cavity. Source: Riedel et al. [59]. (**e**) SEM of a hybrid diamond/silicon-nitride waveguide and a PL map of an NV center within the diamond waveguide. Source: Mouradian et al. [60].

Advances in nanofabrication and photonic design have produced several top-down fabrication solutions to circumventing the diamond-air refractive index mismatch. The solid immersion lens (SIL), consisting of a hemisphere etched around an NV center (Figure 2a), overcomes total internal reflection such that only Fresnel reflection contributes to losses [48,55,56], and the latter can be further reduced using antireflective coatings. When used together with proper orientation of the diamond crystal (Section 4.1), a SIL can increase the saturation count rate to over 1 MCts/s [30,48], resulting in an overall SNR improvement of a factor of five as compared to an NV in a (100)-oriented planar sample. Recently, a metalens constructed from nanopillars etched on the diamond surface was used to image an NV center [57]. In contrast to the SIL, the metalens design collimates the emitted light (Figure 2c), removing the need for a free-space objective and making it a promising approach towards coupling NV centers directly with optical fiber.

An alternative method involves embedding an NV center directly within a diamond pillar or nanowire [61]. The waveguiding effect of the nanopillar directs the emission normal to the diamond surface. An example nanopillar on a [111]-oriented diamond substrate is depicted in Figure 2b [58]. The photons can be collected using an air or oil-immersion objective, with count rates exceeding 1 MCts/s [62]. The nanopillar design has been utilized in improving the sensitivity of scanning magnetometers [63,64]. A related nanophotonic design is the nanobeam [33], which directs emission from embedded NV centers into an underlying substrate and has also yielded saturation count rates > 1 MCts/s. In each of these cases, the high collection efficiency comes at the cost of fabrication complexity, often with the requirement for precise NV alignment relative to the photonic structure. In the case of nanopillars, nanobeams, and other nanophotonic structures that incorporate NV centers close to etched surfaces, detrimental effects from charge and spin noise at the diamond surface further impede performance by reducing the NV center’s optical and spin coherence properties.

### 4.3. Waveguides and Cavities

Integrated single-mode diamond waveguides [65,66] have enabled on-chip optical and spin control of single NVs [60] with saturation count rates approaching 1 MCts/s (Figure 2e). Diamond waveguides can be fabricated using a variety of techniques, with the most common being a diamond-on-insulator approach in which a thin diamond membrane is placed on a lower-refractive-index substrate and patterned using top-down lithography and dry etching [24]. Micro-ring resonators [67] and photonic crystal cavities have been realized in a similar fashion [68,69], both of which exhibit Purcell enhancements (discussed in Section 5) due to the high quality factor of the dielectric cavities.

Due to the relatively small size required for single-mode operation (<300 nm), integrated photonic devices suffer from the same challenges due to fabrication damage and enhanced surface noise as the nanopillar and nanobeam structures discussed in Section 4.2. Furthermore, technical issues associated with submicron diamond membranes (e.g., enhanced strain, nonparallel surfaces and laborious fabrication requirements) have impeded the widespread adoption of these approaches. New designs and fabrication approaches that allow waveguides and cavities to be created directly from bulk diamond crystals [70,71] potentially offer a way forward, although the control of surface noise that causes deteriorated optical linewidths in nanophotonic structures remains a formidable challenge. One approach to avoiding these sources of noise is to use NVs embedded within diamond membranes of micron-scale thickness, which can be aligned within high-finesse fiber-based cavities, albeit with larger mode volumes (Figure 2d) [59,72,73].

### 4.4. Summary

In addition to traditional PL spin readout, every technique described in this review gains performance improvements by increasing the photon collection efficiency. However, constructing optimized structures remains a barrier due to the difficulties associated with nanofabrication of diamond. Detrimental surface effects on the spin and optical coherence properties of shallow NV centers need to be mitigated. Ultimately, in the limit of near-unity collection efficiencies, detector dead times will become a limiting factor to achieving single-shot fidelities, and the use of multiple detectors may be necessary. Overcoming these design, fabrication, materials, and measurement challenges will play a critical role in the development of NV-based quantum devices.

## 5. Radiative Lifetime Engineering

A potential alternative approach to increasing the number of detected photons relies on nanophotonic engineering of the local density of optical states. Dielectric or plasmonic structures can decrease the radiative lifetime and increase the photon emission rate through the Purcell effect [74]. The ability to incorporate quantum emitters within nanophotonic devices has spurred recent efforts to investigate the limits of the Purcell effect, and large gains have been reported [24,75]. The potential for radiative-lifetime engineering to improve the NV center’s optical spin readout efficiency has theoretically been predicted [76], but experimental verification is missing. Since the SNR depends on both the photon count rate and spin contrast (Equation (4)), a better understanding of the optical dynamics in the limit of high Purcell enhancement is required. Here, we provide an overview of current research in this area and highlight several unanswered questions.

Due to their small optical mode volume, dielectric photonic crystal cavities can drastically increase the optical density of states for an embedded NV center [68,69,77,78,79,80]. The cavity not only directs the far-field emission, but also decreases the radiative lifetime by an amount known as the Purcell factor,

(26) $$F_{P}=\frac{3}{4\pi^{2}}{\left(\frac{\lambda }{n}\right)}^{3}\left(\frac{Q}{V}\right),$$

where λ is the free-space wavelength, *n* is the refractive index, *Q* is the quality factor, and *V* is the mode volume. Equation (26) represents the ideal case, assuming a cavity mode resonant with the relevant optical transition and an optical dipole located at the position of maximum field, aligned with its polarization axis. In practice, NV centers can be directly embedded in photonic crystal cavities fabricated from thin diamond membranes [59,68,69,73,80] or positioned close to cavities fabricated in another high-refractive-index material [78,79,81]. The prior method generally results in higher $F_{P}$ than the latter, due to increased spatial overlap between the NV center’s optical dipole and the cavity field [68]. Most investigations have explored how the zero-phonon-line emission around 637 nm can be enhanced [59,68,69,73,78], since photons in this band are ultimately required for coherent spin-photon interfaces with NV centers. Meanwhile, potential effects on the spin-readout SNR for NV centers coupled to photonic crystal cavities remain relatively unexplored.

**Figure 3:**
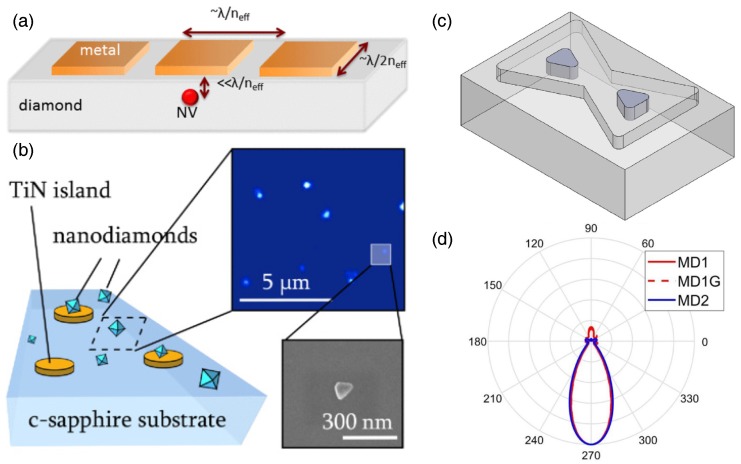
Radiative lifetime engineering with plasmonic devices. Recent examples of plasmonic device geometries include: (**a**) a shallow NV center situated below an optical plasmonic antenna; (**b**) nanodiamonds containing NV ensembles deposited over TiN plasmonic resonators; and (**c**) a hybrid dielectric-metal hourglass structure designed to couple to a shallow NV. Panel (**d**) shows the highly directional angular emission distribution that results from hybrid hourglass plasmonic devices (the device shown in (c) is labeled MD2). “MD” stands for metal-dielectric. See [91] for details on the design variations in (d). Panel (a) is reprinted with permission from [76]. Copyright 2015 by the American Physical Society. Panel (b) is reprinted with permission from [92]. Copyright 2017 by the American Physical Society. Panels (c,d) are from Karamlou et al. [91].

NV centers placed in close proximity to plasmonic resonators can also exhibit large Purcell factors [82,83,84]. The extreme spatial confinement of plasmons can boost $F_{P}$ through a strong reduction of *V* in Equation (26), even when *Q* is generally lower for plasmonic as compared to dielectric structures [85,86]. In fact, a lower *Q* can be desirable for coupling to broadband emission in the NV center’s phonon sideband. As for dielectric cavities, the magnitude of the Purcell enhancement also depends on the relative orientation and location of the optical dipole and the plasmonic mode; at the same time, care must be taken to avoid quenching due to nonradiative energy transfer [87]. The optimal metal-emitter separation depends on the material and geometry; for a gold nanoparticle, the ideal separation is ≈ 5 nm [87], although enhancements have been observed using nanodiamonds with buffers as thick as 30 nm [88]. Figure 3a–c shows three recent examples of plasmonic devices designed to engineer the emission dynamics of NV centers in nanodiamonds or close to the surface of bulk diamond. Several recent studies have further considered metal-dielectric hybrid systems that optimize both directionality and radiative lifetime reduction [89,90,91]. Computational results predict that a hybrid bow-tie structure like the one shown in Figure 3c can produce a strong Purcell enhancement together with highly directional emission (Figure 3d), providing an attractive alternative to all-dielectric diffractive designs.

The question of how Purcell enhancement affects the NV center’s spin-readout SNR remains unresolved. Theoretical studies suggest that substantial improvements in SNR are possible [76,93], but the simulations depend crucially on particular transition rates between excited and ground states that have not been experimentally quantified. The debate centers on how shortening the radiative lifetime influences the PL contrast (see Equations (4) and (25)). Wolf et al. [76] showed that the SNR could increase monotonically with $F_{P}$ if the radiative transitions are fully spin-conserving (such that the overall spin-mixing rate is unaffected by the change in radiative lifetime), whereas only incremental gains in SNR are achievable if the radiative transitions introduce spin mixing that scales with $F_{P}$. A related question concerns the evolution of the NV center’s ground-state spin polarization under optical illumination, which has been predicted to decrease when the radiative rate is enhanced [93]. Recent experiments using NV ensembles within nanodiamonds coupled to plasmonic islands (Figure 3b, [92]) demonstrated that the spin-dependent PL contrast, and subsequently the SNR, decreases with increasing $F_{P}$. This decrease was attributed to additional nonradiative decay pathways present for NV centers in nitrogen-rich nanodiamonds, which ultimately limits the optical excitation rate [94]. The situation is likely to be different for NV centers in higher-purity diamond.

Nanophotonic dielectric and plasmonic structures provide many opportunities to optimize photon emission and electromagnetic coupling properties of NV centers. As discussed further in Section 12, it can be important to consider the ability of such structures to enhance optical absorption in addition to emission. Although the ultimate impact of radiative lifetime engineering on spin readout remains unknown, future studies into the dynamics of Purcell-enhanced NV centers could result in significant improvements to the performance of room-temperature quantum devices.

## 6. Low-Temperature Resonant Readout

The NV center’s triplet excited state is an orbital doublet [95,96]; however, at temperatures above ≈ 20 K, rapid phonon-assisted orbital transitions obscure the fine structure [97], and motional narrowing leads to an effective orbital-singlet excited-state Hamiltonian [98] at room temperature, as shown in Figure 1a. At low temperatures, however, individual spin-selective zero-phonon-line transitions connecting the ground and excited states can be resonantly addressed (Figure 1b), enabling the generation of spin-photon coherence [99,100] and all-optical coherent control of the NV’s orbital and spin dynamics [101,102]. Although this review focuses on room-temperature protocols, in this section we introduce the low-temperature resonance-fluorescence readout protocol, since it offers the highest performance currently available.

In analogous fashion to protocols for resonant optical readout of trapped ions [103] and quantum dot molecules [104], resonance fluorescence allows for single-shot readout of the NV center’s electronic spin state. As initially demonstrated by Robledo et al. [30], the idea is to resonantly pump a spin-selective, spin-preserving optical cycling transition that is protected from the ISC. This improves both the optical contrast and the duration over which photons can be collected. When the external magnetic, electric and strain fields are carefully controlled [105], the $m_{s}=0$ excited states $|E_{x}\rangle$ and $|E_{y}\rangle$ provide nearly ideal cycling transitions, producing PL photons only for the |0〉 spin state. Meanwhile, transitions selective for $m_{s}=\pm 1$ spin states, such as the transition to the $|A_{1}\rangle$ excited state shown in Figure 1c, provide efficient optical pumping pathways to polarize the spin in |0〉 with a 99.7 ± 0.2% probability.

In the initial demonstration [30], resonant readout produced a measurement contrast of 89% persisting for 100 μs. Thresholding provides the best performance in this case; the resulting readout fidelity was 93.2%, corresponding to an SNR improvement by a factor of 34 over the traditional room-temperature PL measurement shown in Figure 1d. Subsequent technical improvements to the resonant readout protocol such as charge stabilization, dynamical stop procedures, and better collection efficiencies have resulted in even higher readout fidelities, enabling the demonstration of quantum feedback [106], heralded entanglement [107], loop-hole free Bell’s inequality violations [20], and quantum error correction [108].

## 7. Nuclear-Assisted Readout

The NV center’s electronic spin can interact with nearby nuclear spins. Prevalent nuclear species include the NV center’s intrinsic nitrogen nuclear spin (with total spin I=1 or $\frac{1}{2}$ for the isotopes ${}^{14}$N and ${}^{15}$N, respectively) and the carbon isotope ${}^{13}$C (total spin $I=\frac{1}{2}$). ${}^{13}$C nuclei are normally present at stochastic locations proximal to the NV center due to its 1.1% isotopic abundance. Nuclear spins exhibit much longer spin lifetimes than electrons [109], and they can be utilized as quantum memories [10,11,13] and computational nodes for quantum error correction [108] and quantum communication [110,111]. In this section, we discuss how coupled nuclear spins can assist in improving readout of the NV center’s electronic spin state [14,112,113].

**Figure 4:**
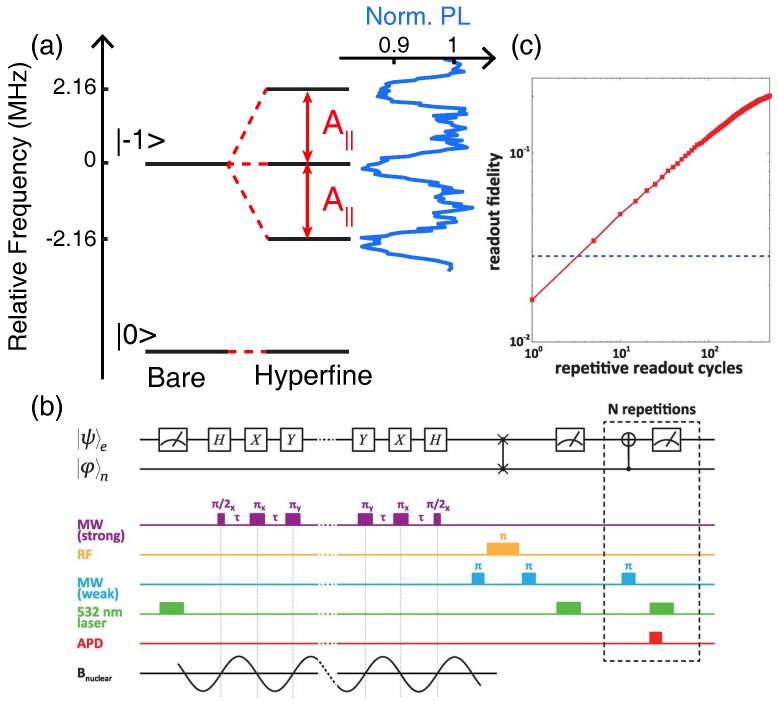
Nuclear-assisted readout. (**a**) Energy-level diagram showing the splitting of the $m_{s}=-1$ spin state into a triplet through hyperfine coupling with ${}^{14}$N ($A_{\left|\right|}=2.16\;\mathrm{MHz}$). The data at the right show the normalized PL response to a pulsed electron-spin resonance measurement. (**b**) Quantum circuit and measurement timing diagram used to detect proteins on the diamond surface using a nitrogen nuclear spin as a memory for storage. (**c**) The readout fidelity, the inverse of spin readout noise (Equation (24)), as a function of repetitive readout cycles. Panels (b,c) are from [17]. Reprinted with permission from AAAS.

The coupling between the NV center electron spin and a single nuclear spin is described by the hyperfine interaction. The hyperfine Hamiltonian can be written in the form:

(27) $${\hat{H}}_{\mathrm{hf}}=A_{∥}{\hat{S}}_{z}{\hat{I}}_{z}+\frac{A_{⊥}}{2}\left({\hat{S}}_{+}{\hat{I}}_{-}+{\hat{S}}_{-}{\hat{I}}_{+}\right),$$

where ${\hat{S}}_{z}$ and ${\hat{I}}_{z}$ are the electron and nuclear Pauli-*z* operators, respectively; ${\hat{S}}_{+/-}$ and ${\hat{I}}_{+/-}$ are the electron and nuclear spin raising and lowering operators, respectively; $A_{∥}$ is the parallel hyperfine component; and $A_{⊥}$ is the perpendicular hyperfine component. The magnitudes of $A_{∥}$ and $A_{⊥}$ depend on the two spin species, their relative orientation, and their separation. Physically, the parallel component represents a nuclear-spin-dependent Zeeman shift of the electron spin eigenstates, clearly observed as a splitting in the electron spin resonance spectrum, as shown in Figure 4a for the case of an intrinsic ${}^{14}$N nuclear spin triplet with $A_{∥}=2.16\;\mathrm{MHz}$. The split resonances will be resolved as long as the hyperfine strength $A_{∥}$ exceeds the electron-spin dephasing rate, $1/T_{2}^{*}$. Such a spectrum allows for the application of nuclear-spin-selective C${}_{n}$NOT${}_{e}$ quantum gates on the electron spin, and likewise electron-spin-selective C${}_{e}$NOT${}_{n}$ gates on the nuclear spin using appropriate radio-frequency driving fields.

The perpendicular component describes flip-flop interactions that mix states with $\Delta m_{s}=-\Delta m_{i}=\pm 1$, causing unwanted electron and nuclear spin flips. For weakly-coupled nuclei under most conditions, flip-flop interactions are suppressed by the large zero-field splitting between electron-spin sub-levels in the NV center’s ground state, and the second term in Equation (27) can be neglected; this is the so-called secular approximation. However, the nonsecular terms are not negligible for strongly-coupled ${}^{13}$C nuclei close to the defect [13], and similarly, the $A_{⊥}$ coupling to intrinsic ${}^{14}N$ and ${}^{15}N$ spins is substantially larger in the NV center’s excited state than in its ground state due to increased overlap with the excited-state electronic orbitals.

The basic idea of nuclear-assisted readout for NV centers, as first demonstrated by Jiang et al. [112], is to harness the long spin lifetime for nuclei and the ability to correlate the electron and nuclear spin states using C${}_{n}$NOT${}_{e}$ gates, such that the PL signal from many successive readout cycles can be accumulated to amplify the SNR. In preparation for measurement, the electron spin state to be measured is mapped onto the nucleus using a series of C${}_{n}$NOT${}_{e}$ and C${}_{e}$NOT${}_{n}$ gates (Figure 4b). The readout then consists of the repeated application of C${}_{n}$NOT${}_{e}$ followed by traditional PL readout of the electron spin. The first readout cycle collapses the nuclear spin into an eigenstate, and ideally, each subsequent cycle polarizes the electron spin but does not affect the nucleus, such that the photon counts from each readout window can be added. In reality, the number of cycles is limited by backaction from the measurement that eventually flips the nuclear spin.

The initial demonstration by Jiang et al. [112] used a ${}^{13}$C nucleus with relatively strong coupling ($A_{∥}=14\;M\mathrm{Hz}$). The map-and-measure procedure was repeated 30 times, improving the SNR by a factor of 2.2 compared to the traditional PL method. Subsequent improvements to the protocol, utilizing a ${}^{15}$N nuclear spin [17], resulted in an overall SNR boost by a factor of 6.8 after 500 cycles (Figure 4c). This readout performance, used together with a sequence of quantum operations on the electron spin designed to sense weak oscillating magnetic fields from nuclear ensembles outside the diamond (Figure 4b), enabled the detection of deuterated proteins on a diamond surface [17].

The nuclear-assisted technique is technically demanding, requiring the application of complex quantum-control pulse sequences at both microwave and radio frequencies, precise alignment of an external dc magnetic field, and the identification or creation of an NV center with a suitably-coupled ${}^{13}$C or ${}^{15}$N (the natural isotopic abundance of ${}^{15}$N is 0.4%). Furthermore, the time required for the C${}_{n}$NOT${}_{e}$ gate scales as $A_{∥}^{-1}$. This gate time introduces substantial overhead in the measurement, especially for weakly-coupled nuclei, limiting the measurement bandwidth and suppressing the sensitivity. On the other hand, more strongly-coupled nuclei suffer from unwanted spin-flips due to the nonsecular terms in Equation (27), limiting the number of cycles that can be performed and the achievable SNR.

For example, the ground-state hyperfine coupling to ${}^{14}$N is only $A_{∥}=2.16\;M\mathrm{Hz}$, and the secular approximation holds (Figure 4a), whereas in the excited state, $A_{∥}\approx A_{⊥}\approx 40\;M\mathrm{Hz}$. Cycling through the excited state is unavoidable during the readout protocol, however, and the $A_{⊥}$ coupling severely limits the nuclear spin lifetime. At room-temperature, the flip-flop probability is maximized at the excited-state level anti-crossing (LAC) near 500 G [114]. Interestingly, flip-flop transitions near the LAC can actually serve to increase the SNR, since a cascaded set of transitions allow for the spin-dependent PL contrast to persist for longer times, leading to a $\sqrt{3}$ increase in SNR [113]. Such cascaded transitions should produce sub-Poissonian noise [115], in which case the achievable SNR improvement might actually be somewhat larger. However, this technique only works within ±50 G of the excited sate LAC, and it requires both electron and nuclear control pulses.

Alternatively, at very high magnetic fields (B>2500 G), the large energy separation of spin eigenstates suppresses flip-flop interactions with ${}^{14}$N, as long as the field is precisely aligned to the NV-center symmetry axis. By operating at these fields, Neumann et al. [14] reached the single-shot readout regime for the ${}^{14}$N nuclear spin, with a fidelity of 92%. Subsequent analysis of the single-shot technique in the context of quantum sensing shows how the time-averaged SNR can be improved by an order of magnitude compared to traditional PL readout [116].

Despite their technical difficulty, nuclear-assisted readout protocols have been widely used in state-of-the-art demonstrations of single-NV quantum sensors [17,18,117,118,119]. Ideally, nuclear-assisted readout demands the following criteria: fast C${}_{n}$NOT${}_{e}$ operations to minimize measurement overhead, minimization of nonsecular components of the hyperfine Hamiltonian, and a nuclear spin with a long lifetime. These criteria are somewhat contradictory, in that fast gate operations require relatively strong coupling, which usually leads to larger nonsecular terms and shorter nuclear lifetimes. Nonetheless, they can be met in practice using any of the common nuclear species: ${}^{14}$N, ${}^{15}$N, or ${}^{13}$C. Application-specific experimental requirements often dominate the final selection. The primary physical limitation in most demonstrations remains the small, but non-zero, electron-nuclear flip-flop probability, especially in the NV center’s excited state. These nonsecular terms can be reduced by selecting coupled ${}^{13}$C, which are closely aligned with the NV-center symmetry axis [120]. For the case of nuclear quantum memories [11], uncontrolled transitions between the negatively- and neutrally-charged states of the NV center present further complications. Continued research into controlling these effects can help to extend the coupled nuclear spin’s lifetime [11,12]. For quantum sensing applications, full consideration of the measurement-duration overhead (Equation (16)) can help to optimize the sensitivity, especially for measurements on faster timescales.

## 8. Spin-to-Charge Conversion

Whereas incomplete PL contrast and spin repolarization limit the fidelity of traditional spin measurements, the NV center’s charge state can be measured optically with high precision, even at room temperature. Given a means for mapping spin projections onto charge populations, or spin to charge conversion (SCC), charge measurements provide an alternate means to boost the spin-readout fidelity. SCC mechanisms are widely used in other spin-qubit platforms including quantum dots [121] and silicon donors [122]. In this section, we review two related mechanisms for all-optical SCC that exploit the NV center’s ISC dynamics, and discuss how the tunability of the subsequent charge-readout process can be an advantage.

High-SNR readout of the NV center’s charge state was first demonstrated for single NV centers by Waldherr et al. [123], and the idea has since been extended to NV ensembles [124,125] and nanodiamonds [94]. The charge readout mechanism depends on the energy difference between the zero-phonon line (ZPL) optical transitions for the neutral (NV${}^{0}$, ZPL at 575 nm) and negative (NV${}^{-}$, ZPL at 637 nm) charge configurations, both of which are stable at room temperature. By selecting an excitation wavelength between these ZPLs, such as 592 nm, only the NV${}^{-}$ configuration is excited. When the optical power is tuned well below saturation, it is possible to detect more than one photon from NV${}^{-}$ before an optically-induced charge transition to the dark NV${}^{0}$ state occurs [45,126].

**Figure 5:**
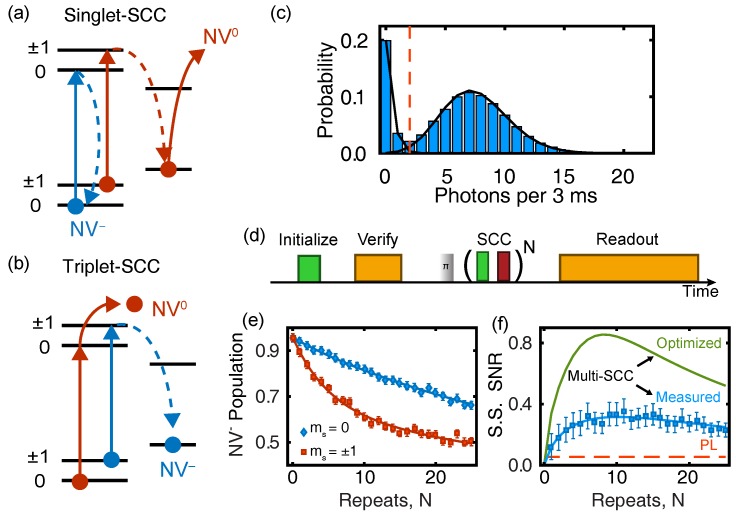
Spin-to-charge conversion (SCC). (**a**,**b**) Schematics of the spin-dependent ionization pathways for singlet spin-to-charge conversion (S-SCC) and triplet-SCC (T-SCC), respectively. Solid lines represent laser induced transitions, while dashed lines represent decay transitions. (**c**) Histogram of photon counts during a 3 ms charge readout measurement with 592 nm illumination [43]. (**d**) Timing diagram for the S-SCC protocol. (**e**) NV${}^{-}$ population for different initial spin states as a function of the number of S-SCC repeats, *N* [43]. (**f**) Single-shot (S.S.) SNR for S-SCC as a function of *N* for the protocol as-demonstrated and for the optimal case assuming 100% singlet ionization probability. The corresponding traditional-PL SNR is the dashed line at SNR = 0.055 [43]. Panels (**c**–**f**) are from [43]. Copyright 2016 by the American Physical Society.

The charge-readout SNR can be varied by changing the excitation power and readout duration. By using low powers and a readout duration >1ms, single-shot charge fidelities exceeding 99% have been demonstrated for single NVs within photonic structures [33,43]. Figure 5c shows an example of the photon-count histogram that results from a 3-ms-duration charge-readout measurement using 592 nm light following initialization with a 532 nm pulse. The clear separation of the count distribution into two Poissonian peaks is characteristic of high-fidelity readout, in this case with F=99.1±0.4% using the threshold of two photons shown by a dashed vertical line.

SCC can be achieved in two related ways, as shown in Figure 5a,b. Both techniques leverage the strong spin selectivity of the ISC from the NV center’s ${}^{3}$E triplet excited state to the singlet manifold. Following a single excitation event, a spin initially in $m_{s}=\pm 1$ crosses to the singlet state with ≈50% probability, whereas the $m_{s}=0$ state undergoes ISC only 5% of the time [41]. Therefore, both techniques begin with a shelving step, consisting of a short, <20 ns, visible pulse of light that excites the triplet manifold with high probability. After waiting for a time longer than the ${}^{3}$E excited-state lifetime (typically ≈ 20 ns), a large fraction of the initial $m_{s}=\pm 1$ spin population is stored in the metastable singlet ground state. Next, an intense ionization pulse resonant with either the singlet absorption band (900– 1042 nm, Figure 5a) or the triplet absorption band (500– 637 nm, Figure 5b) is applied to ionize the singlet or triplet populations, respectively. Hereafter, the methods will be referred to as singlet-SCC and triplet-SCC, depending on which manifold is ionized.

The two methods each have advantages and disadvantages. Triplet-SCC relies on a highly efficient two-photon ionization process for the triplet using ≈600– 637 nm light [33,126]. This can be the same color used for both the shelving step and subsequent charge readout [43], which simplifies experiments. However, the triplet-SCC efficiency is ultimately limited by the 50% ISC probability for $m_{s}=\pm 1$ spin states, since any population that remains in the triplet after the shelving step is ionized. Shields et al. [33] essentially reached the practical limit for this technique, demonstrating a single-shot F=67%, corresponding to an SNR increased by a factor of 4.1 over traditional PL (the SNR ratio in this case is limited by the high collection efficiency in this experiment).

Singlet-SCC, on the other hand, leaves the triplet population unaffected, and the shelve-ionize procedure can be rapidly repeated as shown in Figure 5d, ideally to reach the maximum SCC efficiency given by the ≈10:1 spin-dependent ISC branching ratio. Figure 5e,f shows how the spin-dependent charge contrast and corresponding single-shot SNR vary with the number of repeats, *N*. Drawbacks of this approach include the need for both visible and near-infrared optical beams, and the small optical cross-section for the singlet optical transition [44], which necessitates a high-intensity near-infrared pulse to achieve 100% ionization efficiency. For the data shown in Figure 5e,f, the singlet ionization probability was only 25%, and the singlet-SCC protocol achieved a maximum F=62%, corresponding to an SNR increase by a factor of 5.8 over traditional PL [43]. The infrared pulses used by Hopper et al. [43] were derived from a supercontinuum laser, bandpass filtered to 900– 1000 nm, with an average picosecond pulse energy of 2 nJ. Since the ionization rate depends quadratically on pulse energy, increasing the pulse energy by an order of magnitude should lead to ionization probabilities exceeding 99%. Assuming 100% ionization can be achieved using higher optical pulse energies, Figure 5f shows how the singlet-SCC protocol with N=8 repeats can achieve SNR > 0.84, corresponding to F>75% and an increase over traditional PL by a factor of 15.

Recently, the benefits from SCC have been explored in materials platforms more suited to sensing such as NVs beneath planar surfaces [127], shallow NVs in nanopillars [128], and NV ensembles within type-Ib nanodiamonds [94]. These promising results suggest that SCC can boost the performance of myriad applications.

## 9. Photocurrent Readout

The free electrons and holes produced from photoionization can be utilized as an observable of the NV center’s spin state. By taking advantage of the same spin-dependent ionization phenomenon that enables SCC (see Section 8), spin-dependent photocurrents can be produced. Although it is still in the early stages, electrical readout potentially offers improvements in speed, together with a scalable means for integrating many NV devices on a chip with high density. In this section, we overview the recent advances in photocurrent spin-readout and discuss future directions of research.

Photocurrent readout is possible due to the propensity for the $m_{s}=\pm 1$ spin states to be shelved into the singlet manifold [129], protecting them from ionization for roughly the singlet lifetime (≈200ns). Meanwhile, if optical intensities well above the saturation value drive the triplet optical transition, rapid ionization and recombination processes generate free electrons and holes, respectively, for the initial $m_{s}=0$ spin projection. The goal of photocurrent readout is two-fold: to maximize the number of free carriers produced within the 200 ns shelving time through the use of very high intensity 532 nm illumination, and to efficiently collect and amplify the current while avoiding unwanted noise. Initial experiments demonstrated electrical detection of continuous-wave electron spin resonance for ensembles of NV centers [129,130]. Recent advances in device design, lock-in detection, pulsed measurements, and multi-color pump beams have lead to improved contrast [131,132] and a current detection limit of only five NV centers [133].

Looking forward, the detection of spin-dependent photocurrent from a single NV center remains an outstanding challenge. Such experiments will require careful analysis of the entire electronic noise budget and materials optimization to remove background photocurrents due to substitutional nitrogen and other defects [134]. Due to the similarity with optical SCC, certain aspects of the SCC pulse sequences could be adapted to electrical readout. Systematic investigations of the optimal shelf and ionization colors, durations, and powers could further increase the SNR from photocurrent based readout. Despite these challenges, electrical detection of NV center spin states has enormous potential for developing integrated sensors and devices.

## 10. Accounting for Measurement Overhead

When averaging is required, the time spent initializing and measuring reduces the achievable time-averaged SNR and sensitivity (see Equations (16) and (17), respectively). Since traditional PL readout consists of a short duration of a few hundred nanoseconds, the measurement overhead is usually a fixed penalty with little room for improvement. However, more advanced readout protocols such as low-temperature, nuclear-assisted, and SCC techniques feature measurement times that can be comparable to or longer than typical spin evolution times. In this case, the measurement overhead becomes a major factor, but there is also added flexibility in designing protocols since the single-shot SNR typically depends on the measurement duration. Optimizing the trade-off between the number of experimental repeats and single-shot SNR can result in drastic improvements in time-averaged SNR. Here, we describe the process for optimizing the measurement overhead in the context of SCC readout, using a model that can be directly adapted to nuclear-assisted readout [116] and low-temperature readout.

An arbitrary NV-center measurement can be broken up into three times: the initialization time, $t_{I}$, the wait time, $t_{W}$, and the readout time $t_{R}$, such that the total duration of a single measurement is:

(28) $$\tau =t_{I}+t_{W}+t_{R}.$$

Following from Equation (16), the time-averaged SNR is given by:

(29) $${\langle \mathrm{SNR}\rangle }_{\mathrm{SCC}}=\sqrt{\frac{T}{\tau }}\mathrm{SNR}\left(t_{R},P_{R}\right),$$

where *T* is the total integration time, and $\mathrm{SNR}(t_{R},P_{R})$ is the single-shot SNR as a function of $t_{R}$ and the optical power, $P_{R}$. The single-shot SNR can be experimentally calibrated for various settings of $\left(t_{R},P_{R}\right)$, or it can be calculated using a numerical model of the charge readout process accounting for ionization and recombination processes that become important as $P_{R}$ increases [33]. Given desired experimental settings for $t_{I}$ and $t_{W}$, optimal readout parameters can be chosen to maximize ${\langle \mathrm{SNR}\rangle }_{\mathrm{SCC}}$. In some cases, it can also be beneficial to optimize over $t_{W}$ and $t_{I}$, e.g., for sensing applications by using a suitable formulation for the field sensitivity (Equation (17)) that accounts for the signal amplitude as a function of $t_{W}$, as well as the time-averaged SNR. In general, experiments with longer wait times such as dynamical decoupling sequences for ac field sensing, $T_{1}$ measurements, and controlled interactions with nuclear spins stand to gain the largest performance improvements from SCC.

A useful metric to quantify the SCC performance is the speedup,

(30) $$\mathrm{Speedup}=\frac{T_{\mathrm{PL}}}{T_{\mathrm{SCC}}}=\frac{\tau_{\mathrm{PL}}}{\tau_{\mathrm{SCC}}}{\left(\frac{{\mathrm{SNR}}_{\mathrm{SCC}}}{{\mathrm{SNR}}_{\mathrm{PL}}}\right)}^{2},$$

where $\tau_{\mathrm{SCC}}$, $T_{\mathrm{SCC}}$, SNR${}_{\mathrm{SCC}}$ ($\tau_{\mathrm{PL}}$, $T_{\mathrm{PL}}$, and SNR${}_{\mathrm{PL}}$) represent the measurement-cycle duration, total integration time, and single-shot SNR for SCC (PL), respectively. The speedup quantifies the reduction in total integration time required to achieve a desired time-averaged SNR when using SCC as opposed to traditional PL. A speedup >1 implies that SCC is more efficient than traditional PL. When the time-averaged SNR is optimized over $t_{R}$ and $P_{R}$ as a function of $t_{W}$, the value of $t_{W}$ at which the speedup exceeds unity is referred to as the break-even time.

**Figure 6:**
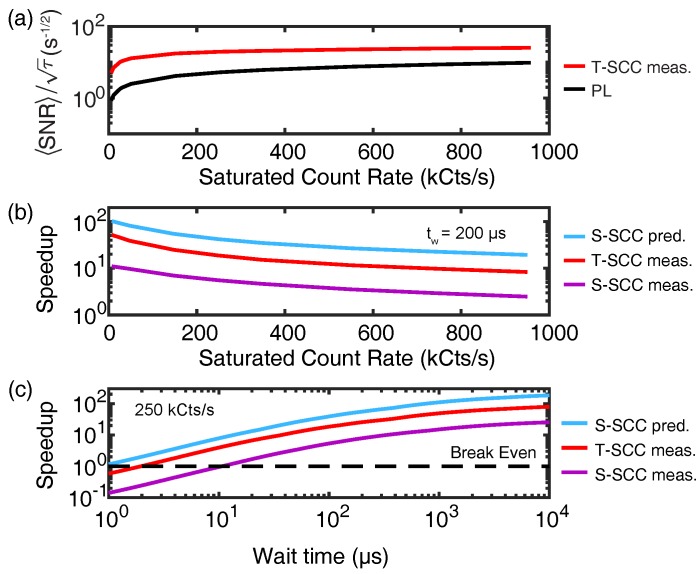
Quantifying SCC improvements in experiments. (**a**) Time averaged SNR scaled by $\sqrt{\tau }$, for the traditional PL and triplet-SCC protocols as a function of saturation green-illumination count rate, assuming $t_{W}=200\;\mu s$. The T-SCC SNR is numerically calculated using the model in [33]. (**b**) Speedup comparison for the various SCC techniques as a function of green saturation count rate, assuming $t_{W}=200\;\mu s$. (**c**) Speedup comparison as a function of $t_{W}$, assuming a green saturation count rate of 250 kCts/s. The dashed line indicates the “break-even” point, where SCC provides a more efficient readout than traditional PL. The speedup in (b,c) is calculated using data reported in [33,43].

All of these quantities need to be calculated or measured for a given experimental setting accounting for the photon collection efficiency, SCC efficiency, etc. Figure 6 gives an example of such optimization calculations, showing how the time-averaged SNR for PL and SCC protocols depend on photon count rate, and the corresponding speedup as a function of the count rate and $t_{W}$. The flexibility of optimizing the SCC readout settings can offer impressive gains; both singlet-SCC and triplet-SCC exhibit order-of-magnitude speedups for experimentally relevant wait times, and the optimized singlet-SCC protocol approaches a 100-fold speedup.

The application of the measurement overhead optimization framework could prove beneficial in drastically speeding up nuclear-assisted and low-temperature readout experiments. Future extensions of this technique could focus on additionally optimizing initialization times, where in-the-loop feedback is used to verify proper charge, nuclear, or electron states.

## 11. Real-Time Signal Processing Techniques

The growing number of spin-readout techniques discussed in the previous sections all aim to overcome photon shot noise by increasing the number of photons that can be recorded in each measurement cycle. In this situation, it is beneficial to leverage signal-processing techniques that make use of the time-of-arrival information of each photon, as opposed to simply summing the total number of detections in a fixed time window. This approach can even be applied to traditional PL spin readout, with an SNR improvement of 7% [42]. Much larger gains can be achieved when each measurement yields multiple photons. Together with low-temperature resonance-fluorescence readout, real-time detection protocols have been essential for the achievement of heralded entanglement [107] and partial measurements [106], since they boost the readout fidelity while reducing unwanted backaction. Similarly, hidden Markov models can improve the performance of room-temperature, single-shot charge-state readout [29]. These results imply that significant improvements should be achievable for room-temperature applications using real-time signal processing in conjunction with nuclear-assisted or SCC readout protocols. With the increasing number of related demonstrations and the larger quantum-information community’s emphasis on open-source tools [135,136], the technological hurdles of implementing real-time analysis will be overcome.

## 12. Discussion

**Figure 7:**
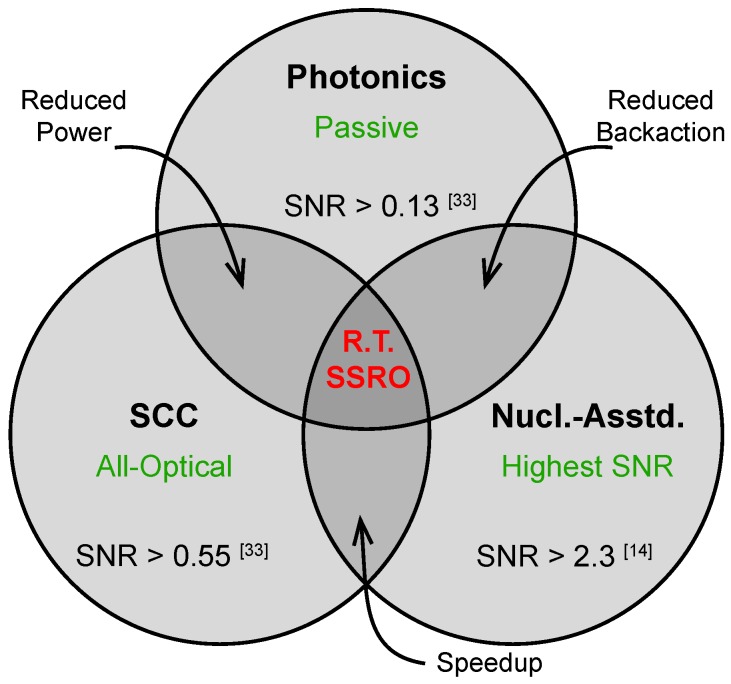
Complementary approaches for enhanced spin readout. Existing techniques have advantages for particular applications. Future research can consider the potential for combining multiple techniques in order to achieve fast, high-fidelity, single-shot readout (SSRO) of the NV center’s electron spin at room temperature. The highest reported traditional PL SNR, as well as the SCC SNR, are from Shields et al. [33]. The highest nuclear assisted SNR is from Neumann et al. [14].

Although the techniques and approaches discussed in this review have mostly developed independently, they are not mutually exclusive. Figure 7 depicts the key advantages of room-temperature approaches based on photonics engineering, SCC, and nuclear quantum logic, including the current state-of-the-art SNR achieved in each case. In many cases, combinations of multiple techniques could overcome existing limitations and provide significant improvements in spin-readout SNR.

In addition to providing a Purcell enhancement of the NV center’s PL emission rate, as discussed in Section 5, plasmonic antennae can also be designed to enhance optical absorption from incident radiation fields. Enhancing absorption is especially important for biological applications, where background autofluorescence and phototoxicity associated with incident 532 nm light limit the optical intensity that can be applied and the achievable SNR. Absorption-enhancing plasmonic structures similar to those currently used to improve thin-film solar cells [137] could reduce the input power, and even enable up-converting schemes for biological applications based on two-photon absorption at near-infrared wavelengths [138]. Similarly, the singlet-SCC protocol would also benefit from absorption enhancements at near-infrared wavelengths, since its fidelity is currently limited by incomplete ionization of the singlet manifold due to a small singlet absorption cross-section [43]. Photocurrent-based readout techniques would also stand to gain from absorption enhancement, due to the high power requirements for rapidly changing of the charge state.

Real-time signal-processing techniques present immediate benefits to applications requiring non-destructive, single-shot readout, and they can also improve the performance of sensing applications. For example, real-time analysis reduces the average measurement time for charge detection by a factor of two [29], and the exact same hardware can verify proper charge-state initialization. Purifying measurements on the basis of the NV${}^{-}$ charge state leads to reduced noise [43,123] and is compatible with sensing schemes [33]. Usually, such purification is performed using post-selection, but a combination of real-time charge verification and dynamic readout should yield more than a 50% improvement in SNR for both SCC and nuclear-assisted readout protocols.

The key limitation on readout fidelity for nuclear-assisted readout protocols is the effective nuclear spin lifetime, which is reduced by cycling between the electron spin’s ground and excited states [14]. The current solution to this problem is to work at very high magnetic fields where hyperfine-induced bit-flip errors are reduced, but radiative lifetime engineering that reduces the excited-state lifetime presents an alternative approach. Achievable radiative rate enhancements of up to two orders of magnitude [91,92,139] could increase the nuclear-spin $T_{1}$ by a similar factor, enabling higher-fidelity measurements or relaxing the field constraints. Alternatively, combinations of nuclear-assisted and SCC readout protocols could take advantage of SCC’s wide measurement tunability to limit the total number of optical cycles while maintaining overall SNR. Either of these approaches could further reduce the readout errors for room-temperature, single-shot readout to the level of a few percent.

## 13. Conclusions

In reviewing the state-of-the-art for optical spin readout of the diamond NV center, we hope to spur further advances in this field and encourage the adoption of more sophisticated techniques in future experiments. In general, enhancements result from increasing the number of detected photons, either by directly modifying the photon emission rate or by mapping the electron spin state onto a longer-lived observable. Each of these approaches has advantages for particular applications, with varying technical requirements in terms of fabrication technology and experimental complexity. For this reason, there is no clear front runner, and it is likely that all of these techniques will continue to improve in future experiments. To date, there has been little exploration into how different techniques can be combined with each other (Figure 7) or enhanced using real-time signal processing. Since spin-readout performance impacts nearly every application of NV centers for quantum science and technology, these questions are compelling avenues for future research.

## Figures and Tables

**Table 1:** Compilation of spin-readout metrics, their formal relation to differential SNR, and common use cases. PL, photoluminescence; SNR, Signal-to-noise ratio.

Metric	Relation to SNR	Use Case
Contrast, *C*, & Count rate, $\alpha_{0}$	$\mathrm{SNR}=\sqrt{\alpha_{0}}\frac{C}{\sqrt{2-C}}$	traditional PL readout
Spin-readout noise, $\sigma_{R}$	$\mathrm{SNR}=\sqrt{\frac{2}{\sigma_{R}^{2}-1}}$	magnetometry
Fidelity, F	$\mathrm{SNR}=\frac{p_{0|0}-p_{0|1}}{\sqrt{p_{0|0}\left(1-p_{0|0}\right)+p_{0|1}\left(1-p_{0|1}\right)}}$	quantum algorithms, large signals
Repeats for 〈SNR〉=1	$N={\left(\frac{1}{\mathrm{SNR}}\right)}^{2}$	magnetometry, general experiments

## Abbreviations

The following abbreviations are used in this manuscript:

NV	Nitrogen-Vacancy
PL	Photoluminescence
SNR	Signal-to-noise ratio
SCC	Spin-to-charge conversion
ISC	Intersystem crossing
LAC	Level anti-crossing
SQL	Standard quantum limit
ZPL	Zero-phonon line

## Appendix A. Spin-Readout Noise Calculations

Following Equation (11), in order to calculate the spin-readout noise, $\sigma_{R}$, we must identify the signal’s dependence on spin-projection angle, $\frac{\partial \langle S\rangle }{\partial \theta }$, and the standard deviation, $\sigma_{S}$. The standard deviation differs from the common form for Poisson or binomial random variables due to the signal consisting of the sum of two random variables with different weights, and therefore, we use the general expression for the variance,

(A1) $$\sigma_{S}^{2}=\langle S^{2}\rangle -{\langle S\rangle }^{2}.$$

In this Appendix, we derive expressions for $\sigma_{R}$ corresponding to photon summation and thresholding signals.

### Appendix A.1. Photon Summation

For photon summation, the signal (Equation (10)) directly reflects the number of detected photons:

(A2) $$\langle S\rangle =\cos^{2}\left(\frac{\theta }{2}\right)\alpha_{0}+\sin^{2}\left(\frac{\theta }{2}\right)\alpha_{1}.$$

From this expression, we can directly derive the signal variation,

(A3) $$\frac{\partial \langle S\rangle }{\partial \theta }=\frac{1}{2}\sin\left(\theta \right)\left(\alpha_{0}-\alpha_{1}\right),$$

and the expectation value of the signal squared,

(A4) $$\langle S^{2}\rangle =\cos^{2}\left(\frac{\theta }{2}\right)\left(\alpha_{0}^{2}+\alpha_{0}\right)+\sin^{2}\left(\frac{\theta }{2}\right)\left(\alpha_{1}^{2}+\alpha_{1}\right).$$

The latter expression uses the fact that the expected value of $X^{2}$ from a Poisson distribution P(X;λ) with mean-value λ is $\langle X^{2}\rangle =\lambda^{2}+\lambda$. By combining Equations (A1)–(A4) with Equation (24), we arrive at the following general expression for the spin-readout noise:

(A5) $$\sigma_{R}^{\mathrm{Photon}}=\frac{\sqrt{\frac{1}{4}\sin^{2}\left(\theta \right){\left(\alpha_{0}-\alpha_{1}\right)}^{2}+\cos^{2}\left(\frac{\theta }{2}\right)\alpha_{0}+\sin^{2}\left(\frac{\theta }{2}\right)\alpha_{1}}}{\frac{1}{2}\sin\left(\theta \right)\left(\alpha_{0}-\alpha_{1}\right)}.$$

Assuming an equally-weighted superposition state (θ=π/2), Equation (A5) reduces to the form reported in Equation (22) of the main text.

### Appendix A.2. Thresholding

In the case of thresholding, the signal results from the binary values of the measurement, where we assume that S=1 corresponds to the identification of the zero spin state (Equation (6)), and therefore:

(A6) $$\langle S\rangle =\cos^{2}\left(\frac{\theta }{2}\right)p_{0|0}+\sin^{2}\left(\frac{\theta }{2}\right)p_{0|1}.$$

As before, we use this expression to calculate the signal variation,

(A7) $$\frac{\partial \langle S\rangle }{\partial \theta }=\frac{1}{2}\sin\left(\theta \right)\left(p_{0|0}-p_{0|1}\right),$$

and the mean of the signal squared,

(A8) $$\langle S^{2}\rangle =\cos^{2}\left(\frac{\theta }{2}\right)p_{0|0}+\sin^{2}\left(\frac{\theta }{2}\right)p_{0|1}.$$

These expressions yield the following general form for the spin-readout noise associated with thresholding,

(A9) $$\sigma_{R}^{\mathrm{Threshold}}=\frac{\sqrt{{\left(\cos^{2}\left(\frac{\theta }{2}\right)p_{0|0}-\sin^{2}\left(\frac{\theta }{2}\right)p_{0|1}\right)}^{2}+p_{0|0}\left(\cos^{2}\left(\frac{\theta }{2}\right)-2\cos^{4}\left(\frac{\theta }{2}\right)\right)+p_{0|1}\left(\sin^{2}\left(\frac{\theta }{2}\right)-\sin^{4}\left(\frac{\theta }{2}\right)\right)}}{\frac{1}{2}\sin\left(\theta \right)\left(p_{0|0}-p_{0|1}\right)},$$

Assuming an equally-weighted superposition state (θ=π/2), Equation (A9) reduces to the form reported in Equation (23) of the main text.
